# Construction and analysis of sample-specific driver modules for breast cancer

**DOI:** 10.1186/s12864-022-08928-4

**Published:** 2022-10-20

**Authors:** Yuanyuan Chen, Haitao Li, Xiao Sun

**Affiliations:** 1 State Key Laboratory of Bioelectronics, School of Biological Science and Medical Engineering, Southeast University, Nanjing, 210096 P. R. China; 2 College of Science, Nanjing Agricultural University, Nanjing, 210095 P. R. China

**Keywords:** Sample-specific driver module, 2-order network theory, Sample-specific network method, Synergistic collaboration, Common driver pattern

## Abstract

**Background:**

It is important to understand the functional impact of somatic mutation and methylation aberration at an individual level to implement precision medicine. Recent studies have demonstrated that the perturbation of gene interaction networks can provide a fundamental link between genotype (or epigenotype) and phenotype. However, it is unclear how individual mutations affect the function of biological networks, especially for individual methylation aberration. To solve this, we provided a sample-specific driver module construction method using the 2-order network theory and hub-gene theory to identify individual perturbation networks driven by mutations or methylation aberrations.

**Results:**

Our method integrated multi-omics of breast cancer, including genomics, transcriptomics, epigenomics and interactomics, and provided new insight into the synergistic collaboration between methylation and mutation at an individual level. A common driver pattern of breast cancer was identified from a novel perspective of a driver module, which is correlated to the occurrence and development of breast cancer. The constructed driver module reflects the survival prognosis and degree of malignancy among different subtypes of breast cancer. Additionally, subtype-specific driver modules were identified.

**Conclusions:**

This study explores the driver module of individual cancer, and contributes to a better understanding of the mechanism of breast cancer driven by the mutations and methylation variations from the point of view of the driver network. This work will help identify new therapeutic combinations of gene mutations and drugs in humans.

**Supplementary Information:**

The online version contains supplementary material available at 10.1186/s12864-022-08928-4.

## Introduction

Cancer is generally thought to be driven by the continuous accumulation of somatic mutations throughout the life cycle of an individual, as well as by epigenetic and transcriptional changes [1]. Gene regulation is closely related to the development of cancer. Gene regulation of cell growth can be divided into two types. The first is responsible for maintaining the growth of the body's cells at a constant rate of renewal, and the second is responsible for slowing or stopping cell growth so that new cells can take over [2].

Some gene mutations will directly or indirectly lead to uncontrolled cell growth, which could promote cancer progression. These kinds of genes are called driver genes. However, some genes with mutations will not cause cell growth, and will not lead to the development of cancer. These mutation genes are called passenger genes [3–5]. Mutations in driver genes confer selective advantages on tumor cells, such as an increased ability to divide, which allows cells to evade apoptosis, reproduce endlessly, evade the immune system and other defenses, spread and invade other tissues, and alter the environment for their benefit [3]. It is important to identify driver genes to investigate the drive mechanism of cancer development. There are many methods to identify driver genes, which are categorized into five types based on their major features [6]: mutation frequency-based methods, functional impact-based methods, structure genomics-based methods, networks- or pathways-based methods, and data integration-based methods. Currently, the most complete panorama of cancer driver genes contains 568 driver genes, collected on the integrative OncoGenomics (IntOGen) platform [7].

Early cancer research mainly focused on genetics, specifically on the activation of proto-oncogenes or the inactivation of tumor-suppressor genes [3–5]. However, since the 1990s, more and more studies have recognized that the regulation of epigenetic variation is crucial in the occurrence and progression of human cancer. A study by researchers at the USDA/ARS Children's Nutrition Research Center shows that epigenetic changes alone can cause cancer [8]. Epigenetic variations are present in almost all human cancers and are now known to collaborate with genetic mutations to drive cancer phenotypes [9]. However, there are few methods of methylation-driven gene identification. Differentially methylated genes that affect gene transcription were identified as methylation-driven genes by comparing the methylation status with that of normal tissues in the MethylMix algorithm, which was designed by the Stanford University Biomedical Information Center [10].

Cancer is highly heterogeneous, and there are differences in genotype and phenotype between different patients [11]. Therefore, it is necessary to clarify the sample-specific molecular mechanism of cancer and formulate individualized treatment plans according to the personalized characteristics of each patient. In addition, genes do not exist in isolation but interact with each other to form an organic biological network [12, 13]. A biomolecular interaction network can represent biological processes more reliably. By integrating multi-omics data, a large number of network methods have been proposed. Variable selection methods have played a major role in integrating multi-omics data [14] and the networks of omics features can be identified through variable selection. For example, Wu et al. have constructed networks by robustly integrating multi-platform omics features and using penalization to accommodate the high dimensionality [15]. Similarity network fusion is another method that can integrate multiple omics data and optimize visualization results [16]. It constructs a sample similarity network for each of the data types (such as mRNA expression data, DNA methylation, and image data), and then iteratively integrates these networks using a novel network fusion method. These network methods using multi-omics integration account for the heterogeneity of cancer data.

In the process of cancer occurrence and development, mutations or methylation aberrations will disturb the interactions between genes, affecting the orderly biological system and the phenotype of the patient. The perturbation of gene interaction networks connects the cancer genotype and epigenotype to the phenotype [17]. As such, the construction of the individual specific gene interaction perturbation network is extremely important for explaining the cancer mechanism driven by the mutations and methylation aberrations. This work integrates somatic mutation data, gene expression data, methylation and gene interactions network data to identify the sample-specific mutation and methylation aberration driver modules (i.e., cancer-related perturbed biological network driven by mutation or methylation aberration genes) using a multi-omics data mining method.

## Materials and methods

### Data sources

#### Gene expression data

The RNA-Seq data in the form of Fragments Per Kilobase Million (FPKM) and clinical data of breast cancer, including 1097 breast cancer samples and 113 tumor-adjacent breast tissue samples, were downloaded from The Cancer Genome Atlas (TCGA) database (http://tcga-data.nci.nih.gov/tcga/ accessed on 24 June 2020) using TCGA-Assembler 2 (version 2.0.6, http://www.compgenome.org/TCGA-Assembler/). Additional clinical data of all samples were downloaded using the R package RTCGA (version 1.22.0). The expression data of breast cancer samples and tumor-adjacent breast tissues were assigned to a case group and reference group, respectively. The downloaded expression matrices were directly used as inputs in our method.

#### Somatic mutation data

Genome-wide nonsynonymous somatic mutations on 18,847 significantly mutated genes of 986 breast cancer tumors in TCGA were downloaded from UCSC Xena (version 07–18-2019, https://tcga-xena-hub.s3.us-east-1.amazonaws.com/download/mc3_gene_level%2FBRCA_mc3_gene_level.txt.gz). Only nonsynonymous mutations were used for the following analysis.

#### Methylation data

The DNA methylation data (level three) in TCGA generated on the HM450 platform including 485,577 total probes [18], were downloaded from the UCSC Xena (version 07–19-2019, https://gdc-hub.s3.us-east-1.amazonaws.com/download/TCGA-BRCA.methylation450.tsv.gz) for 794 breast cancer samples and 96 tumor-adjacent breast tissue samples. The methylation level of each probe was measured by its beta value, which ranges from 0 (unmethylated) to 1 (completely methylated). The CpG probe annotation file was downloaded from the UCSC Xena database (https://gdc-hub.s3.us-east-1.amazonaws.com/download/illuminaMethyl450_hg38_GDC).

#### Background network

Synthetic lethality (SL) is a phenomenon that was first observed in drosophila melanogaster experiments more than 100 years ago. Synthetic lethality is a type of genetic interaction between two genes where the simultaneous perturbations of the two genes will result in cell death or a dramatic decrease in cell viability, though a perturbation of either gene alone is not lethal [19].

In the saccharomyces cerevisiae experiment, most of the genes were found to have this SL effect with each other, and the SL relationship between these genes could also be applied to human tumor genes [20]. Passenger mutations that do not directly contribute to tumorigenesis, as well as mutations that disrupt cellular networks and promote a cancerous state of cells, could be candidates for SL cancer therapies. A novel comprehensive map of synthetic-lethal interactions between genes that are mutated in cancer may have significant clinical potential [21]. Multiple studies have indicated that SL is a promising form of gene interaction for cancer therapy, and can identify specific genes to target cancer cells without disrupting normal cells [22, 23]. In addition, an SL will help functionally interpret the vast number of mutations identified in cancer genome sequencing studies [21].

Consequently, the SL interactions built by Jing et al. from the Synthetic Lethality genes interactions Database (SynLethDB) [24], which consists of 6513 genes and 19,955 synthetic lethal gene pairs for human tumors, were downloaded as the background Network (v1.0, the last accessed date, 20–04-2021, http://synlethdb.sist.shanghaitech.edu.cn/#/).

### Overview of the sample-specific driver module construction approach

**Fig. 1:**
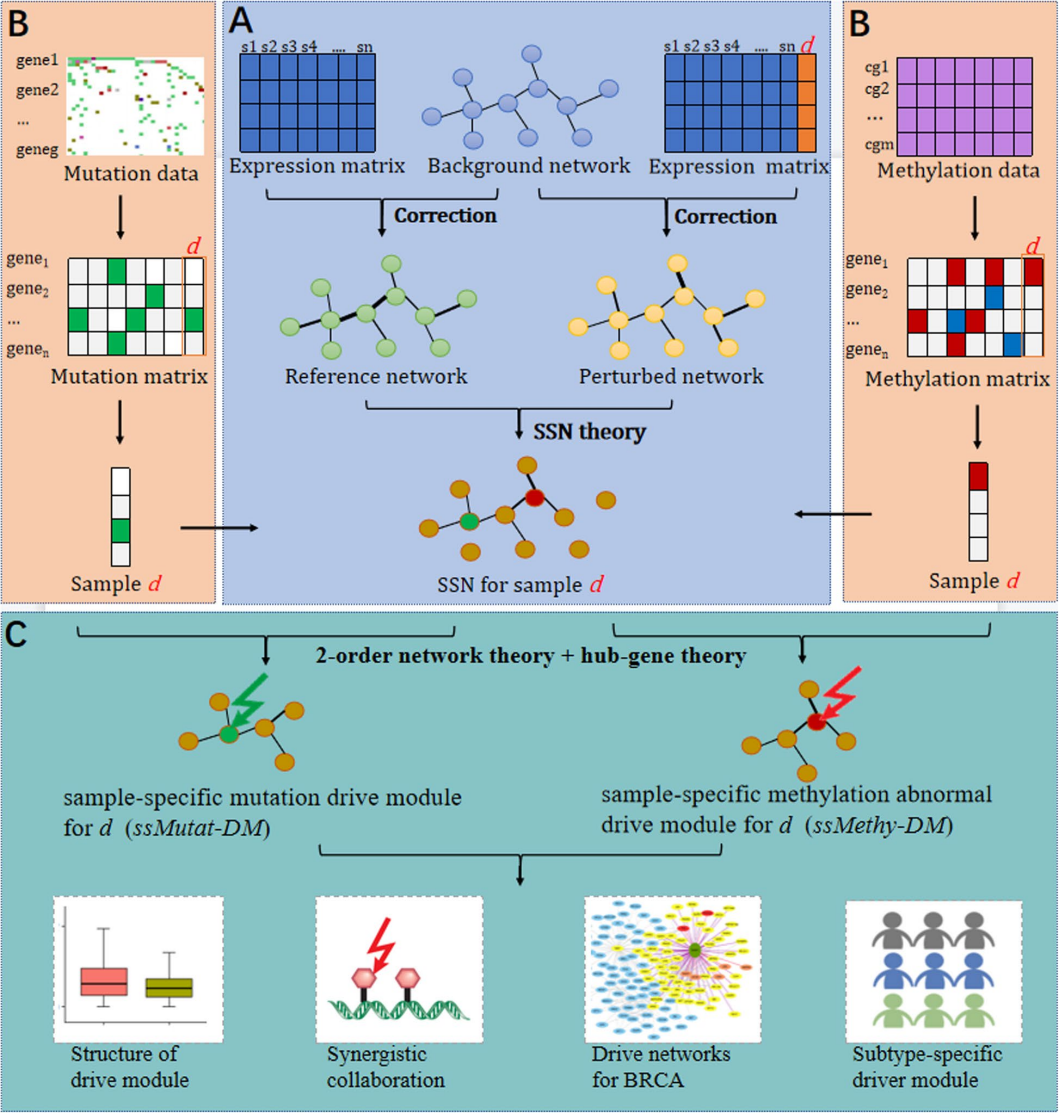
Flowchart of the three-step procedure used to identify the sample-specific driver module. **A** The sample-specific network (SSN) for each breast cancer sample was constructed based on the expression matrix and gene interaction network. For a group of reference samples (n samples), a reference network was first constructed by the correlation analysis. A new sample d was added to the group, and the perturbed network with this additional sample was constructed based on the combined expression data. The SSN for sample d was constructed by using the SSN theory. **B** The somatic mutation profile for each breast cancer sample was constructed using the somatic mutation data. The methylation aberration profile for each breast cancer sample was calculated by the outlier detection method of the Hampel filter based on the methylation data. **C** The sample-specific mutation driver module (ssMutat-DM) and the sample-specific methylation aberration driver module (ssMethy-DM) for each breast cancer patient were constructed based on the 2-order network theory and hub-gene theory. Subsequent analysis of the driver modules was performed including the structure of driver modules, a synergistic collaboration of mutation and methylation, identification of the common pattern of driver modules for breast cancer, identification of subtype-specific driver modules, and encoding of the functional consequences of mutations and methylation aberrations

We constructed sample-specific driver modules in three steps (see Fig. 1). First, the sample-specific network (SSN) for each breast cancer sample was constructed based on the gene expression profiles and background network by the SSN method introduced in [28]. Second, the somatic mutation profiles of all breast cancer samples were constructed by the somatic mutation data. In addition, based on the methylation data we obtained the aberrantly methylated genes for each tumor sample using the outlier detection method of the Hampel filter, which make up the methylation aberration profiles for breast cancer samples. Finally, the sample-specific mutation driver module (ssMutat-DM) and the sample-specific methylation aberration driver module (ssMethy-DM) were constructed using the 2-order network theory and hub-gene theory.

### Construction of the sample-specific network

In the following, the SSN for each breast cancer sample was constructed based on gene expression profiles by using the SSN method introduced in reference [25] (Fig. 1A). First, using the gene expression profiles of n normal samples, (all tumor-adjacent breast cancer tissues), the reference network was constructed by calculating the correlation coefficient ${\mathrm{pcc}}_{\mathrm{n}}$(the Pearson correlation coefficient (PCC)) of each gene pair connected in the background network. The weights of the edges in the reference network are the PCC of the corresponding gene pairs. Then, the perturbed network of the single breast cancer sample d was constructed by calculating the new correlation coefficient ${\mathrm{pcc}}_{\mathrm{n}+1}$ using the expression profiles of all the reference samples and sample d. The difference between the reference and perturbed network of d is due to the expression profile of sample d. The differential network for the single breast cancer sample d was constructed by the differential correlation coefficients of the corresponding edge between the reference and perturbed networks in terms of PCC:

$${\Delta \mathrm{pcc}}_{\mathrm{n}}={\mathrm{pcc}}_{\mathrm{n}+1}-{\mathrm{pcc}}_{\mathrm{n}}$$

for each edge. In reference [25], Liu et al. have demonstrated that ${\Delta \mathrm{pcc}}_{\mathrm{n}}$ follows a normal distribution with a mean value of 0 and a variance of $(1-{{\mathrm{pcc}}_{\mathrm{n}}}^{2})/(\mathrm{n}-1)$, when n is large enough. The significance level of each edge (${\Delta \mathrm{pcc}}_{\mathrm{n}}$) was calculated by the two-tailed Z-test (or the U-test). The SSN for sample d is constituted by those edges with significant edges (*P*-value < 0.01). The robustness of the SSNs results against the different choices of the reference samples and different reference sample sizes on breast cancer data from TCGA in [25]. These ensure the stability and reproducibility of the sample-specific driver modules.

### Construction of the mutation and methylation matrices

The somatic mutation matrix and the methylation matrix can be constructed by the somatic mutation data and the methylation data (see Fig. 1B).

We first constructed somatic mutation profiles of all cancer samples. Each patient was represented as a profile of binary $(0, 1)$ states on genes, where rows represented genes and columns corresponded to cancer samples. The elements ${\mathrm{M}}_{\mathrm{ij}}$ in the matrix was defined as follows:

$${\mathrm M}_{\mathrm{ij}}=\left\{\begin{array}{c}1,\mathrm{if}\;\mathrm{gene}\;\mathrm i\;\mathrm{mutates}\;\mathrm{in}\;\mathrm{sample}\;\mathrm j,\\\\0,\;\mathrm{if}\;\mathrm{gene}\;\mathrm i\;\mathrm{does}\;\mathrm{not}\;\mathrm{mutate}\;\mathrm{in}\;\mathrm{sample}\;\mathrm j.\end{array}\right.$$

For the methylation matrix, we identified the aberrantly methylated genes for each cancer sample. First, probes with ‘NA’ values among > 10% samples were removed, and we imputed the remaining ‘NA’ values using the 10-nearest neighbors imputation procedure with the ‘impute.knn’ function in the R package ‘impute’ [26]. The information in the CpG probe annotation file includes the corresponding gene and the genomic region, among others. Using the median of the methylation values [27] of the CpG probes mapped at promoter regions (including 1500 bp upstream to the transcription start site TSS1500; 200 bp upstream to the transcription start site TSS200; 5’UTR; and 1stExon), a single methylation value was consolidated for each gene in the HM450K platform. We used the outlier detection method of the Hampel filter to identify the aberrantly methylated genes (i.e., hypermethylation and hypomethylation genes) for each sample. Specifically, each patient was represented as a profile of ternary $(-1, 0, 1)$ states on genes, where rows represented genes and columns corresponded to cancer samples. The elements ${\mathrm{MT}}_{\mathrm{ij}}$ in the methylation matrix were defined as follows:

$${\mathrm{MT}}_{\mathrm{ij}}=\left\{\begin{array}{ll}-1,\;\mathrm{represents}\;\mathrm{hypomethylation},\;&\mathrm{if}\;\mathrm v\;<\;\mathrm{MED}\;-\;3\;\mathrm{MAD},\\\\1,\;\mathrm{represents}\;\mathrm{hypermethylation},\;&\mathrm{if}\;\mathrm v\;>\;\mathrm{MED}\;+\;3\;\mathrm{MAD},\\\\0,\;&\mathrm{otherwise},\end{array}\right.$$

where $\mathrm{v}$ was the methylation value of gene i in patient j, $\mathrm{MED}$ and $\mathrm{MAD}$ were the median and median absolute deviation of methylation value of gene i in all samples, respectively.

### Construction of the sample-specific driver module

The ssMutat-DM and ssMethy-DM were constructed according to the following two theories. The first is the 2-order network theory [28], which states that genes with mutation or methylation aberrations could affect both their neighbors and their neighbors’ neighbors. The second is the hub-gene theory. Model biology studies have demonstrated that hub genes (genes with a high degree in a biological network) have extremely important biological functions, and also play important role in the regulation of other genes in related pathways [29, 30]. In the gene regulatory network related to human disease, hub genes tend to be cancer drivers. Based on the above two theories, we used two principles to select the subnetwork in the SSN for each breast cancer sample: i) the 2-order subnetwork centered on mutation genes, ii) the mutation genes’ neighbors and their neighbors’ neighbors should be hub genes, where the hub genes were regarded as the top 20% of genes with higher degrees in the SSN. The subnetwork was then considered to be the ssMutat-DM for the breast cancer patient, and edges in ssMutat-DM were considered to be mutation-driven edges. Similarly, we constructed the ssMethy-DM for each patient by centering on aberrantly methylated genes (see Fig. 1C). The union of the ssMutat-DM and ssMethy-DM constituted the sample-specific co-driver module (ssCo-DM) for each breast cancer sample.

### Structural analysis of the driver modules

To measure the importance of different genes in the driver modules, the degree, betweenness and eigenvector centrality of genes in the constructed driver modules were analyzed. Degree centrality was determined by the number of connections. Betweenness centrality is a measure of the importance of a node by assessing the number of shortest paths through it. Eigenvector centrality is a measure of a node by assessing the importance of its adjacent nodes in a network. In addition, the similarity of ssMutat-DM and ssMethy-DM of each breast cancer sample was measured by the Jacobi similarity as follows:

$$\mathrm{Similarity}\;\mathrm{score}\;({\mathrm S}_{\mathrm{Mutat}},{\mathrm S}_{\mathrm{Methy}})=\frac{\left|\cap({\mathrm S}_{\mathrm{Mutat}},{\mathrm S}_{\mathrm{Methy}}\right)\vert}{\left|\cup({\mathrm S}_{\mathrm{Mutat}},{\mathrm S}_{\mathrm{Methy}}\right)\vert},$$

where ${\mathrm{S}}_{\mathrm{Mutat}}$ and ${\mathrm{S}}_{\mathrm{Methy}}$ represent the gene set or edge set of ssMutat-DM and ssMethy-DM, respectively.

### Identifying the driver modules for breast cancer and its subtypes

A Monte Carlo simulation test was used to evaluate whether a specific edge was significantly included in the driver modules of more breast cancer samples. This process was repeated 10,000 times and 10,000 edges were selected randomly each time. We derived the statistical significance of the frequency of occurrence in the ssMutat-DM of all breast cancer samples based on empirical NULL distribution generated by 10,000 random shuffling. A cutoff of *P*-value = 0.05 was obtained. All edges with a frequency greater than the mean of the cutoff values made up the mutation driver module for breast cancer. Similarly, we constructed the methylation aberration driver module for breast cancer.

Furthermore, the driver modules for the PAM50 subtypes of breast cancer were analyzed. We computed the edge number of driver modules for each subtype of breast cancer, and the differential analysis for the edge numbers of different subtypes was performed using the Kruskal–Wallis sum test. In addition, pairwise comparisons between subtypes used the Nemenyi test. Edges that are present in more than 60% ssCo-DMs of breast cancer subtype samples constituted the subtype-specific driver module. The unique genes involved in each subtype-specific driver module were used to perform pathway enrichment analysis by Metascape (http://metascape.org). The pathways with *P*-values less than 0.01 were retained. Finally, the subtype-specific pathways were identified.

### Statistical analysis

Unless stated otherwise, comparisons of two groups on one variable were determined using a Wilcoxon rank sum test with continuity correction, two-sided or one-sided, unpaired or paired, as appropriate, and multiple group comparisons using a Kruskal–Wallis rank sum test. Results with *P* < 0.05 were considered significant. Statistical analyses were performed with R (version 4.05) The Pearson correlation coefficients used for the construction of SSNs were calculated by parallel operation on the server with 48 cores.

## Results

### The constructed driver modules

Statistical analysis of the proportion of aberrantly methylated genes in each sample demonstrated that the proportion of genes with alteration methylation is significantly higher in breast cancer samples than in normal samples (see Figure S1A). In addition, the hypermethylation frequency and hypomethylation frequency for each gene were calculated for breast cancer samples. The result shows that the hypermethylation frequencies are significantly higher than those of hypomethylation (*P*-value < 2.2e-16, Wilcoxon rank sum test with continuity correction, see Figure S1B), i.e., methylation aberrations in the promoter region of breast cancer samples tend to be hypermethylation.

**Fig. 2:**
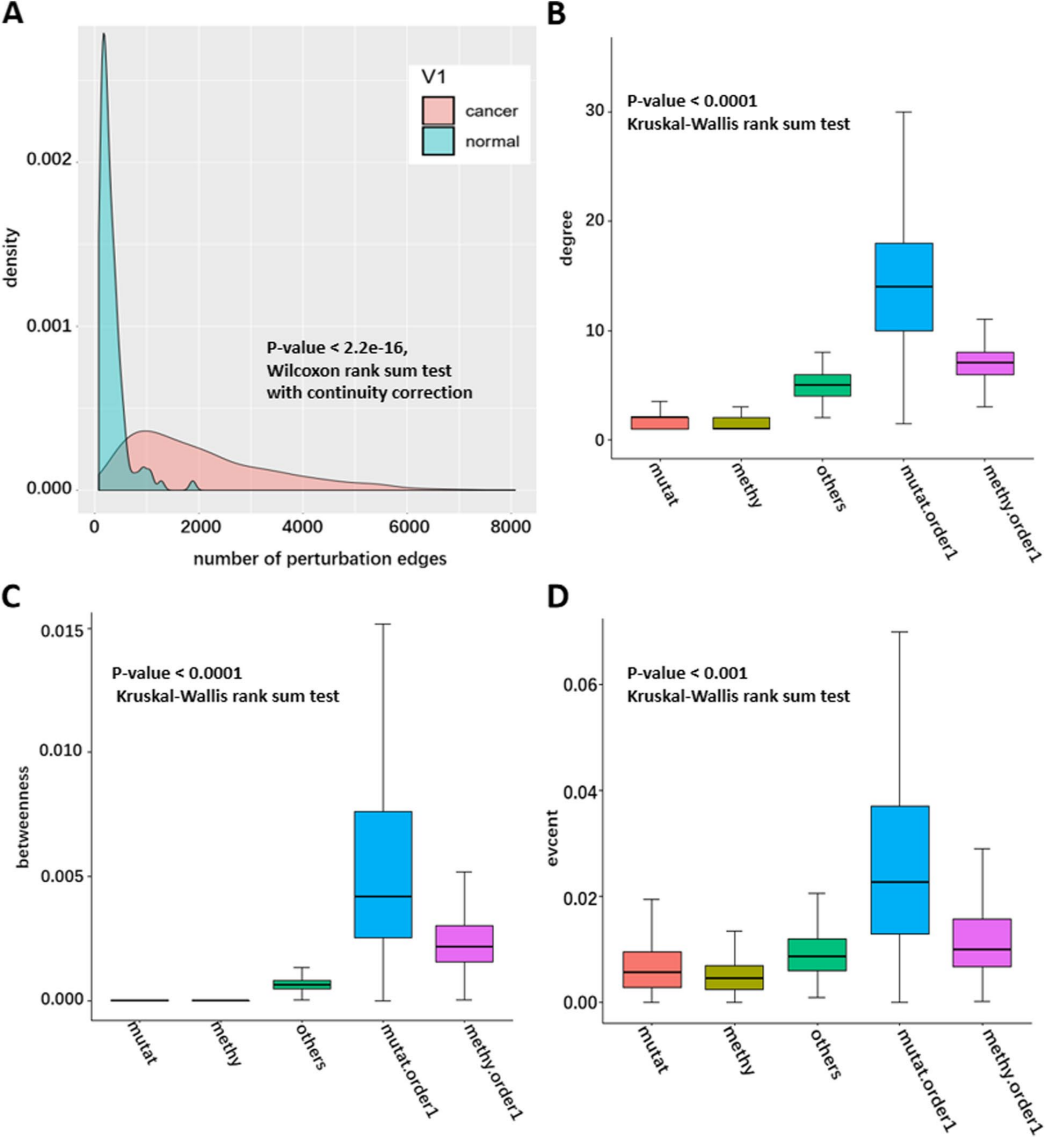
The neighbors of mutated or aberrantly methylated genes are the key genes in the networks. **A** The probability density plot of the edge number in SSN for breast cancer samples and normal samples. **B**, **C**, and **D**, The degree, betweenness and eigenvector centrality distribution of five different groups of genes (including mutation genes, aberrantly methylated genes, other genes besides these two classes, 1-order neighbor of mutation genes, and 1-order neighbor of aberrantly methylated genes) in ssCo-DM

The SSN was constructed for all samples using the above method (see the section on the construction of the sample-specific network for details). The probability density distribution of the edge number in SSN for breast cancer samples and normal samples is shown in Fig. 2A. The number of edges in SSN for each breast cancer sample is significantly greater than that in normal samples (*P*-value < 2.2e-16, Wilcoxon rank sum test with continuity correction), that is, the gene co-expression perturbations in breast cancer samples are more serious.

Each breast cancer sample has a ssCo-DM. The degree of distribution of five different kinds of genes (including mutation genes, aberrantly methylated genes, other genes besides these two classes, 1-order neighbor of mutation genes, and 1-order neighbor of aberrantly methylated genes) in ssCo-DM is shown in Fig. 2B. The degree of mutation genes and aberrantly methylated genes was significantly lower than that of others, but the degree of their neighbors was significantly greater (*P*-value < 0.0001, Kruskal–Wallis rank sum test). In addition to the degree of genes, a similar result was found in the betweenness and eigenvector centrality of the above five kinds of genes in ssCo-DM (see Fig. 2C and Fig. 2D). This indicates that mutation genes or aberrantly methylated genes affect their neighbors, which could be the key genes with important regulatory functions in the networks.

### Synergistic collaboration between methylation and mutation at the individual level

**Fig. 3:**
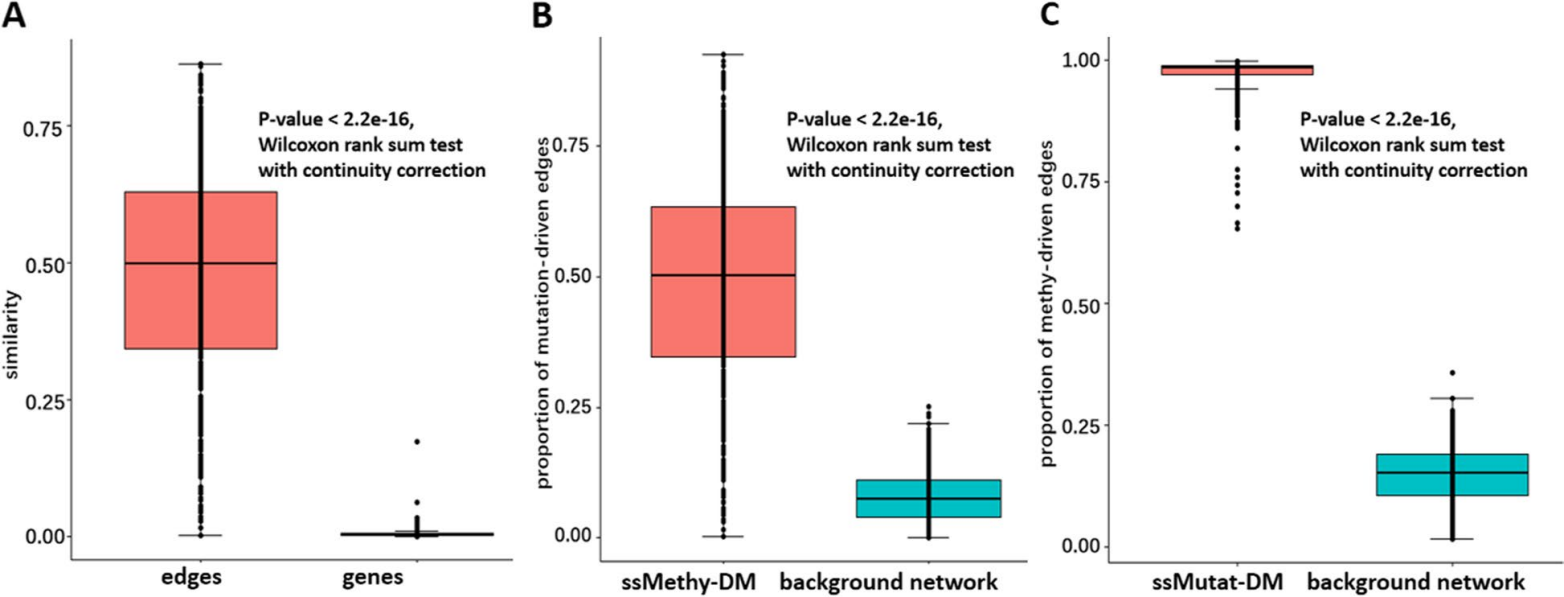
Synergistic collaboration between methylation and mutation at the individual level from the network perspective. **A** The Jacobi similarity of ssMutat-DM and ssMethy-DM in terms of edges, and the mutation genes and aberrantly methylated genes for each sample. **B** The proportion distribution of mutation-driven edges in ssMethy-DM for each breast cancer sample and that in the background network respectively. **C** The proportion distribution of methylation-aberration-driven edges in ssMutat-DM and that in the background network, respectively

The Jacobi similarity of ssMutat-DM and ssMethy-DM in terms of edges, and the mutation genes and aberrantly methylated genes for each sample were computed. The boxplot in Fig. 3A shows that the similarity between the aberrantly methylated genes and the mutation genes is extremely low in each sample, but the similarity between the ssMethy-DM and the ssMutat-DM is significantly higher (*P*-value < 2.2e-16, Wilcoxon rank sum test with continuity correction).

For each breast cancer sample, the mutation frequency and methylation aberration frequency were calculated respectively. The boxplot in Fig. S2A shows that there is no significant difference in mutation frequency between genes with methylation aberration and all other genes, and Fig. S2B also shows that there is also no significant difference in methylation aberration frequency between genes with mutation and all other genes.

This means that genes with alteration methylation have no tendency to mutate at the individual level. Similarly, the boxplot in Fig. S2B shows that mutation genes have no tendency to be methylation aberration either.

We computed the proportion of edges in the mutation driver module per sample to the edges in the overall background network, and the proportion of mutation-driven edges in the methylation aberration driver module for each breast cancer sample. Figure 3B shows that the proportion of mutation-driven edges in the methylation aberration driver module is significantly greater than that in the background network (*P*-value < 2.2e-16, Wilcoxon rank sum test with continuity correction), that is, edges in the methylation aberration driver module tend to be mutation-driven. Conversely, edges in the mutation driver module also tend to be methylation-alteration-driven (see Fig. 3C). This indicates that the interaction could exist between mutation and methylation.

The above results indicate that the synergistic relationship between methylation and mutation at the individual level of breast cancer can be explained from the network perspective but not the gene perspective.

### The common patterns of driver modules for breast cancer

**Fig. 4:**
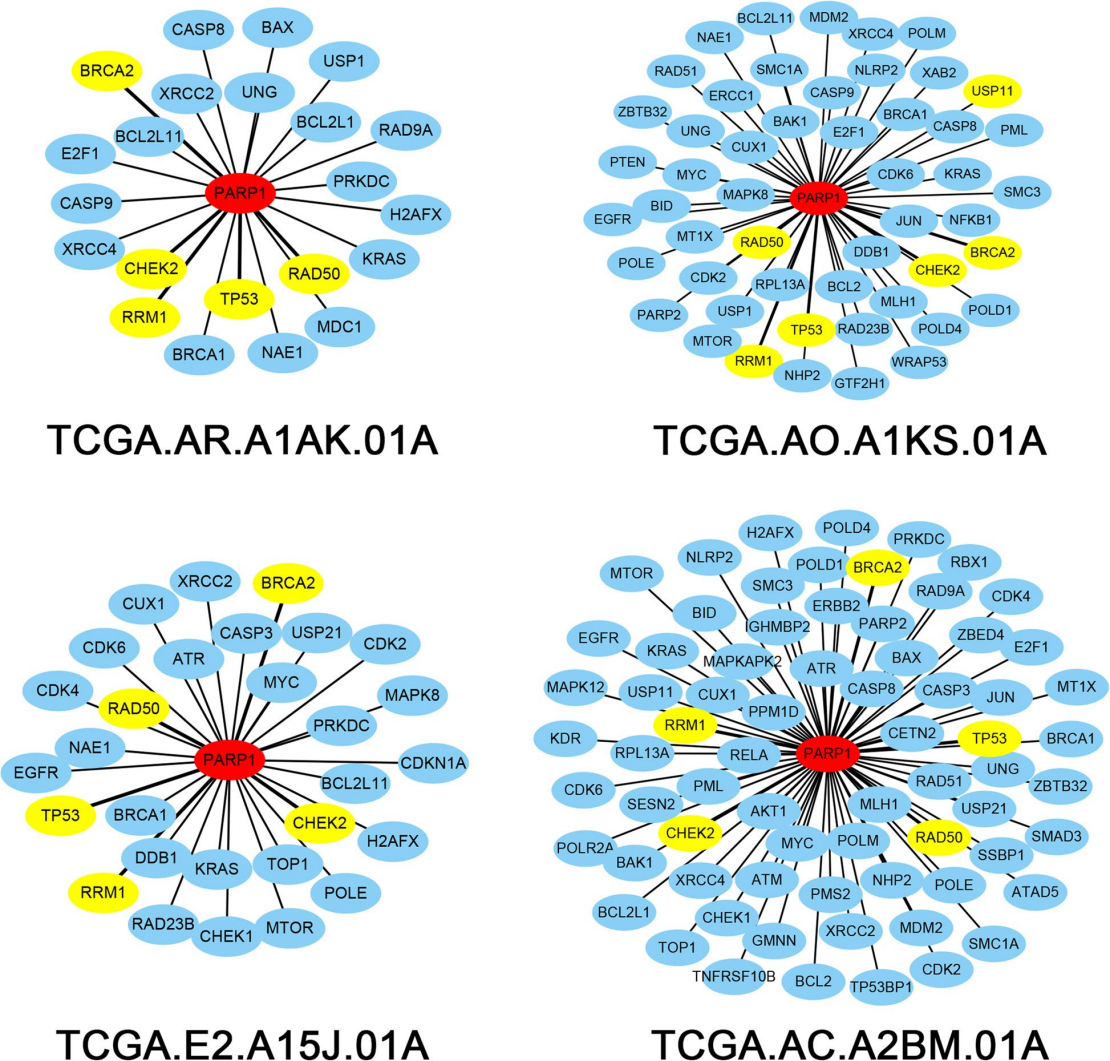
Sample-specific mutation driver modules characterize personalized features and reveal common network patterns for breast cancer. The four sample-specific mutation driver subnetworks of gene PARP1 from four samples of breast cancer are displayed here. The numbers of the connections with PARP1 for the four samples are respectively 23, 52, 30 and 72, and the genes linked to PARP1 are differ across the four breast cancer samples. However, TP53, RRM1, RAD50, CHEK2 and BRCA2 (the yellow color) are common genes appearing in the four subnetworks

To show the power of ssMutat-DM to characterize the personalized features from a network viewpoint, we analyzed the subnetworks of ssMutat-DM related to gene PARP1. The subnetwork of PARP1 is composed of genes directly connected to PARP1. Figure 4 shows four subnetworks of four breast cancer samples, which clearly characterize personalized features. However, the connections between PARP1 and genes TP53, RRM1, RAD50, CHEK2, and BRCA2 exist in all four subnetworks. Actually, we found that 66% of breast cancer samples include a connection between PARP1 and TP53, 70.14% of breast cancer samples include a connection between PARP1 and RRM1, and more than 50% of breast cancer samples include a connection between PARP1 and the other 3 genes RAD50, CHEK2, and BRCA2. These results show that these connections are the common network pattern for breast cancer related to PARP1. The five genes TP53, RRM1, RAD50, CHEK2, and BRCA2 are closely related to the occurrence and development of breast cancer. In addition, similar results could be found in the subnetworks of ssMethy-DM from the four breast cancer samples in Fig. S3.

**Fig. 5:**
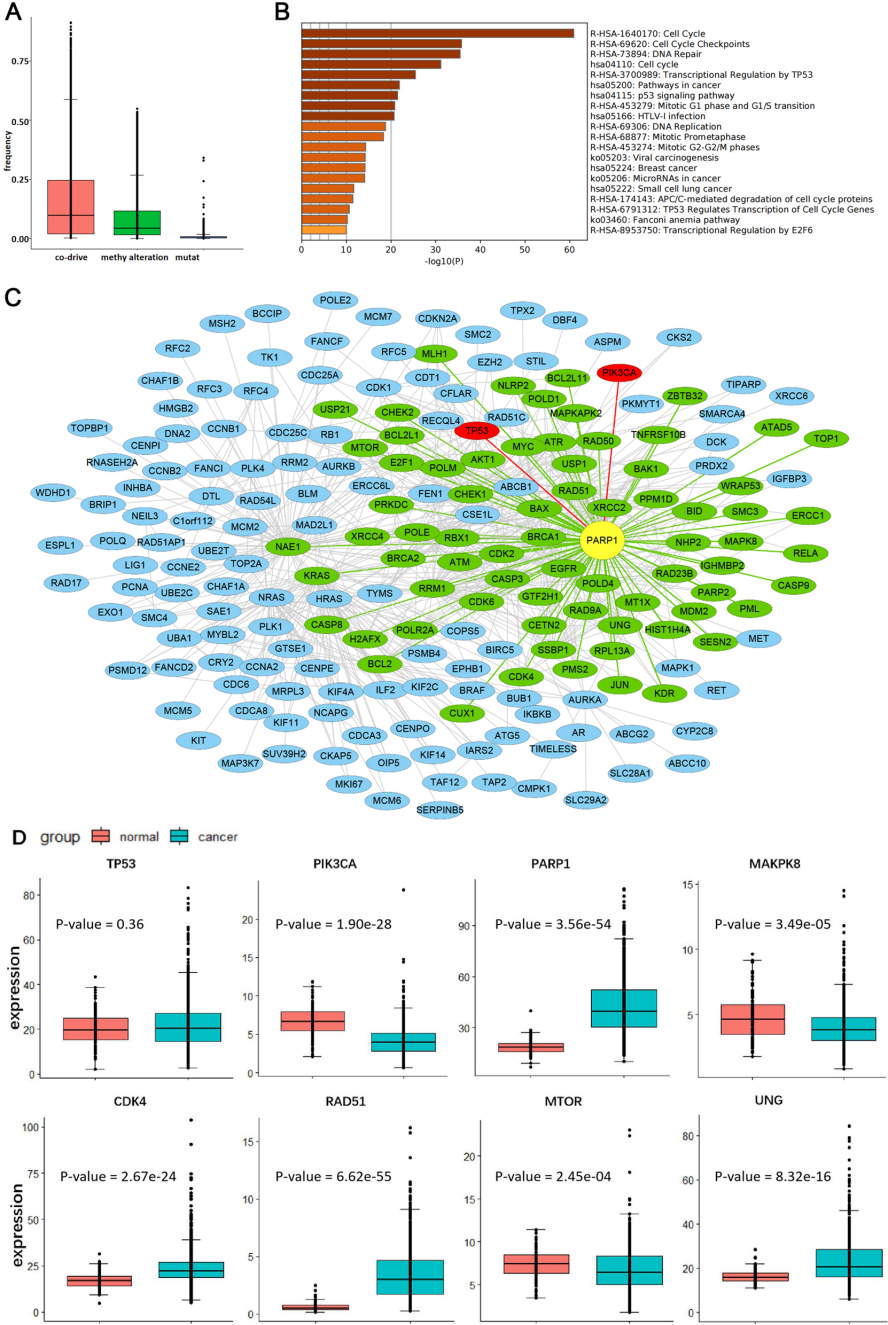
The mutation driver module for breast cancer is closely related to its occurrence and development. **A** Edge co-driver frequency, gene methylation aberration frequency, and gene mutation frequency in breast cancer. **B** The enriched pathways for genes in the network are constructed by edges with a co-driver frequency of more than 75%. **C** The mutation driver module for breast cancer. **D** The expressions of these TP53, PIK3CA, PARP1, MAPK8, CDK4, RAD51, MTOR, and UNG between normal and tumor samples. The *P*-values were obtained by the Wilcoxon rank sum test with continuity correction

Edge co-driver frequency (i.e., the frequency of occurrence of edges in ssCo-DM), gene methylation aberration frequency, and gene mutation frequency are shown in Fig. 5A. There are few genes with mutation frequency or methylation aberration more than 0.5, so it is difficult to identify the common pattern of breast cancer from the perspective of the mutation gene. However, there are 214 edges with a co-driver frequency of more than 75%, which indicates that it could be possible to find the common occurrence and development pattern of breast cancer from the perspective of the network. All of the 117 genes in the network constructed by the 214 edges were used for pathway enrichment analysis. Most of the enriched pathways were closely related to breast cancer (see Fig. 5B), such as cell cycle (checkpoint), pathway in cancer, transcriptional regulation by TP53, P53 signaling pathway, and breast cancer.

The mutation driver module for breast cancer is composed of 736 edges and 203 genes (Fig. 5C). The two genes with the highest mutation frequency in breast cancer are TP53 and PIK3CA.There are only two genes that are connected to both of them in the mutation driver module for breast cancer: CASP3 and PARP1. CASP3 is a hub gene in the mutation driver module for breast cancer, which is involved in the signaling pathways of apoptosis, necrosis, and inflammation [31]. Some studies have shown that the down-regulated expression of CASP3 could represent an important cell survival mechanism in breast cancer and overexpression in breast cancer cells exerts an independent adverse effect on patients' overall survival [32]. And PARP1 is a hub gene with the second-highest degree in the mutation driver module for breast cancer. Indeed, PARP1 inhibitors are currently one of the most promising therapeutic agents for several cancers, especially breast cancer, and can selectively target tumor cells with BRCA1 or BRCA2 tumor suppressor gene mutations through synthetic lethality [33, 34]. In the mutation driver module for breast cancer, both BRCA1 and BRCA2 are exactly connected to PARP1. Furthermore, the expressions of TP53, PIK3CA, and PARP1, as well as 5 random neighbor genes of PARP1 are shown in Fig. 5D. The expressions of these 8 genes are significantly different between tumor and normal samples, except TP53. In fact, most of the neighbor genes’ expression of PARP1 (64/72) are significantly different in tumor and normal tissues per Wilcoxon rank sum test with continuity correction (*P*-value < 0.05).

**Fig. 6:**
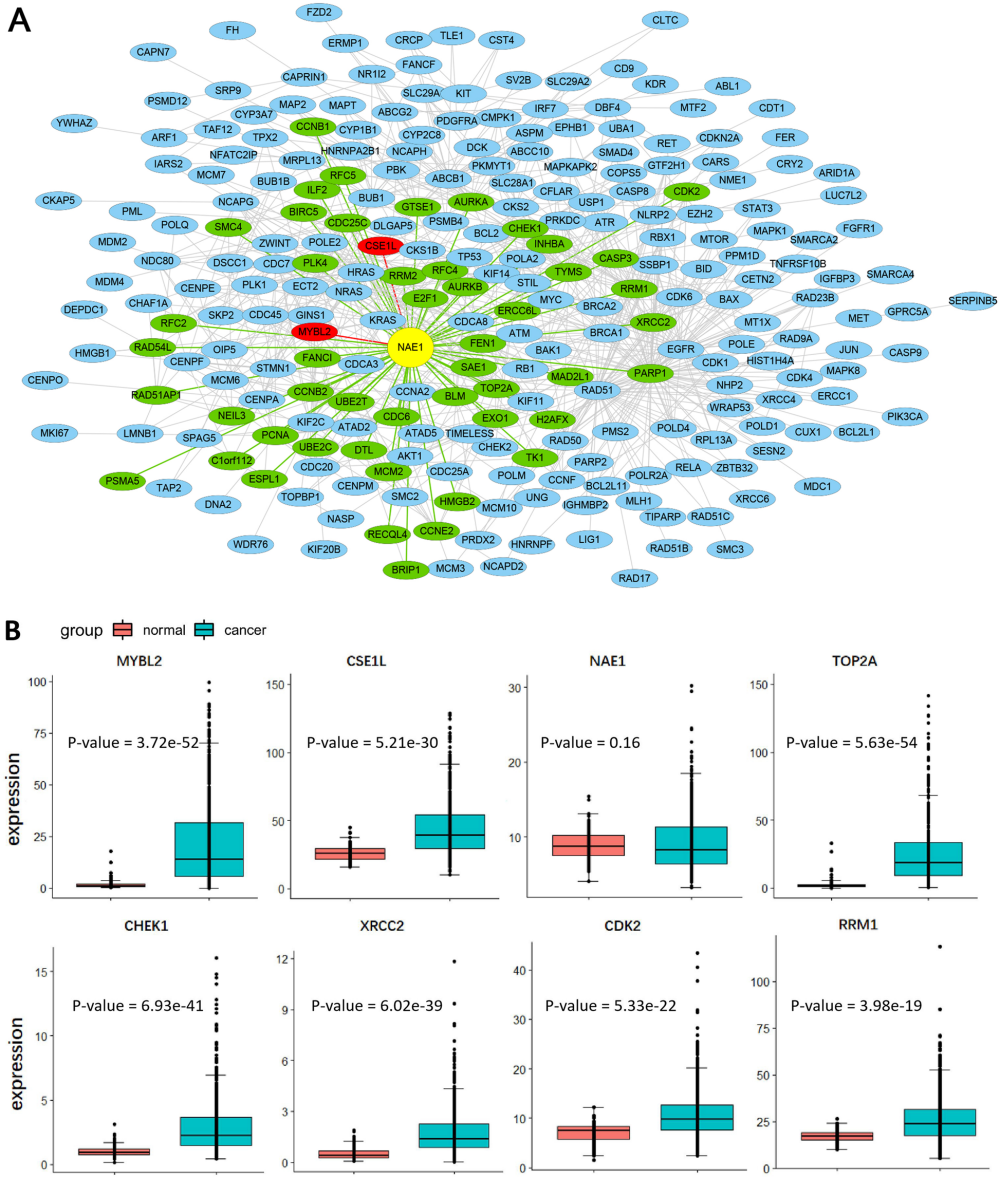
The methylation aberration driver module for breast cancer is related to its occurrence and development. **A** The methylation aberration driver module for breast cancer. **B** The expressions of these MYBL2, CSE1L, NAE1, TOP2A, CHEK1, XRCC2, CDK2 and RRM1 between normal and tumor samples. The *P*-values were obtained by the Wilcoxon rank sum test with continuity correction

The methylation aberration driver module for breast cancer is composed of 799 edges and 257 genes (see Fig. 6A). Similarly, genes MYBL2 and CSE1L with high methylation aberration frequency in breast cancer are both connected to NAE1 with high degree. It is interesting that, the expressions of MYBL2, CSE1L, and most neighbors of NAE1 (59/63) are significantly different between tumor and normal tissues (*P*-value < 0.05, Wilcoxon rank sum test with continuity correction), but as a link gene NAE1 is not differentially expressed (see Fig. 6B).

The above results indicate that mutated or aberrantly methylated genes could affect their 2-order neighbors through 1-order neighbors, which could explain the 2-order network theory.

### The subtype-specific driver modules for breast cancer

**Fig. 7:**
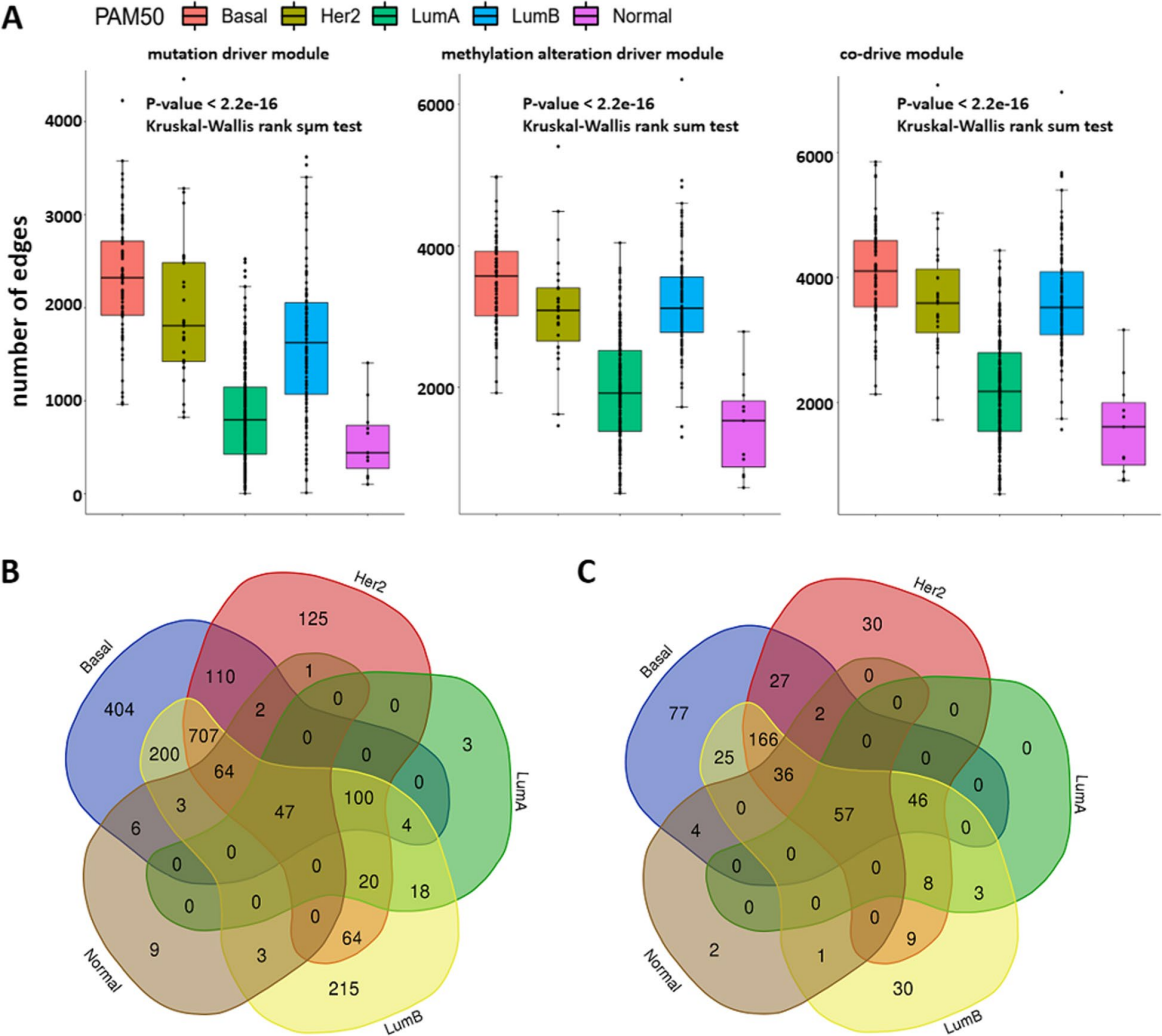
The subtype-specific driver modules for breast cancer reveal the degree of malignancy among different subtypes. **A** The edge numbers of ssMutat-DM, ssMethy-DM and ssCo-DM among PAM50 subtypes of breast cancer. **B** The overlapping of edges in the subtype-specific driver modules. **C** The overlapping of genes in the subtype-specific driver modules

The edge numbers of three kinds of driver modules are significantly different among PAM50 subtypes of breast cancer by the Kruskal–Wallis rank sum test (*P*-value < 2.2e-16, Fig. 7A). Obviously, the Basal-like subtype with a poor prognosis has the most mutation-driven edges in ssMutat-DM, while subtypes of LumA and Normal-like have relatively few mutation-driven edges.

Both LumA and Normal-like are usually correlated with a low degree of malignancy and a good prognosis. Similar results can be found both in ssMethy-DM and ssCo-DM. Table S1 shows the result of pairwise comparisons for the number of edges in subtype-specific driver modules between subtypes. This means that the size of driver modules is closely related to the degree of malignancy and prognosis of breast cancer among subtypes. It is unclear where this is due to the number of mutated or aberrantly methylated genes in different subtypes. Figure S4 shows that the Basal-like subtype does not have significantly more mutation genes than other subtypes. There is no significant difference in the number of aberrantly methylation genes between Basal-like and Normal-like subtypes (*P*-value = 0.8367, Wilcoxon rank sum test with continuity correction). Other pairwise comparisons for the number of mutation genes (or aberrantly methylation genes) between subtypes are displayed in Table S2. Therefore, the number of somatic mutations and aberrantly methylated genes does not explain the heterogeneity and malignancy of breast cancer subtypes well. However, the driver module can reflect the survival prognosis and degree of malignancy among different subtypes. The larger the sample-specific driver module is, the worse the survival prognosis will be.

Overlapping of genes and edges in the subtype-specific driver modules is shown in Fig. 7. There are 47 edges shared by all subtypes, and there are 404, 215, 125, 9, and 3 subtype-specific edges in Basal-like, LumB, Her2, Normal-like, and LumA subtypes, respectively. There are 57 subtype-shared genes, which were used to perform gene enrichment analysis. The enriched pathways are closely related to breast cancer, such as cell cycle, breast cancer, transcriptional regulation in TP53, and signaling by NOTCH. Subtype-specific pathways are shown in Table 1. Signaling by interleukins, signaling by WNT, apoptosis, and VEGF signaling pathway are enriched in Basal-like subtypes. FCERI mediated MAPK activation, and mTOR signaling pathway are enriched in LumB subtype.

**Table 1:** Subtype-specific pathways

**Pathways**	**Basal-like**	**LumB**	**Her2**
Cellular responses to stress	√		
Signalingby Interleukins	√		
Signaling by WNT	√		
Apoptosis	√		
VEGF signaling pathway	√		
Diseases of signal transduction by growth Factor receptors and second messengers	√		
FCERI mediated MAPK activation		√	
Signaling by Receptor Tyrosine Kinases		√	
mTOR signaling pathway		√	
Tight junction		√	
Focal adhesion		√	
Antigen processing: Ubiquitination & Proteasome degradation			√

## Discussion

After accounting for the high heterogeneity and the large sample size of breast cancer data [35–37], we provided a sample-specific driver module construction method and applied it to analyze breast cancer data in TCGA. This multi-omics analysis method decodes the function of somatic mutations and methylation aberrations from the point of view of the co-expression perturbation network at the individual level. In this study, the mutation driver module and methylation aberration driver module for each breast cancer sample were constructed and analyzed, and the synergistic collaboration mechanism between methylation and mutation of breast cancer at the individual level was explored. The common driver pattern for breast cancer was identified from the point of view of the driver module. This driver pattern is closely correlated to the occurrence and development of breast cancer. The constructed driver module could reflect the survival prognosis and degree of malignancy among different subtypes of breast cancer. Finally, we identified subtype-specific driver modules and decoded the function of mutation and methylation in the context of subtype-specific networks. This work, as an exploratory study on the driver module of individual cancer, reveals the mechanism of the occurrence and development of individual cancer, explaining the complex (epi) genotypic-phenotypic relationship of cancer, assisting clinical decision-making, discovering drug combinations, and promoting the development of precision medicine.

Our method is based on the 2-order network theory and hub gene theory, which is a powerful method for identifying the sample-specific mutation and methylation aberration driver modules. We applied our method to breast cancer and found that methylation aberrations in the promoter region of breast cancer samples tend to be hypermethylation, which coincides with previous research about methylation and cancer [38–40]. The synergistic collaboration between methylation and mutation was shown from the perspective of a sample-specific drive network. The common driver patterns for breast cancer were identified, and these driver patterns are correlated to the occurrence and development of breast cancer. We identified the gene PARP1 with the second highest degree in the mutation driver module for breast cancer. Indeed, PARP inhibitors are currently one of the most promising therapeutic agents for breast cancer [39, 40]. Even more interesting, we found that the constructed driver module could reflect the survival prognosis and degree of malignancy among different subtypes of breast cancer. In addition, the identified subtype-shared and subtype-specific driver modules were enriched in pathways related to cancer. These results indicate that our driver module construction method provides an effective framework for the functional characterization of mutations and methylation aberrations in cancer at the individual level.

A growing body of research shows that network-based features are more effective and robust compared to single-gene features when analyzing noisy high-throughput data [41]. Our method is based on the recently proposed concept of ‘edgotype’ and can therefore complement gene-based methods [42]. In addition, different from the perturbation network identified method in [43], our method considers the heterogeneity of individual cancer patients. However, many sample-specific network construction methods are only based on gene expression profiles [29, 44], but ignore the information about mutation and methylation. Based on a samples-specific co-expression perturbation network, our method can identify sample-specific mutation and methylation aberration driver modules by integrating them with the mutations or methylation aberrations of the individual patient. Therefore, this study will improve our understanding of the functional consequences of mutations and methylation aberrations by network perturbations and promote the development of personalized precision medicine.

Our driver module construction method can be extended in several ways. For example, our experiment was carried out on breast cancer, but our method can be applied to other types of cancer. Cancers of different tissues have high heterogeneity, so the application of our method to pan-cancers could be used to explore how different cancers yield different perturbation networks and decode the function of mutations and methylation aberrations in the context of pan-cancer. This could help us to better understand the different occurrence and development mechanisms of different cancers from the perspective of the driver module. Second, although this study only considers mutations and methylation aberrations within genes, other genomic and epigenomic alterations are also observed in cancer. These genetic alterations, such as gene fusions, alternative splicing and copy number variation, are related to the perturbation of pathways [45–47]. Moreover, our method was performed on a synthetic lethality genes interaction network for identifying driver modules. It can be extended to other regulatory networks, such as protein–protein interaction networks, signaling pathways, transcription regulatory networks, miRNA-gene regulatory networks, and so on.

To summarize, we propose a new method that can identify sample-specific mutation or methylation aberration driver modules. This method can be used to characterize the malignant degree and heterogeneity of each patient by the driver network. It is also an exploratory method for analyzing the function of cancer mutations and methylations with the eventual goal of implementing personalized or precision medicine.

## Supplementary Information

**Additional file 1: Supplementary Figure S1.** The identified aberrantly methylated genes for each sample. **Supplementary Figure S2.** The boxplot of gene mutation frequency and gene methylation aberration frequency. **Supplementary Figure S3.** Sample-specific methylation aberration driver modules characterize personalized features and also reveal common network patterns for breast cancer samples. **Supplementary Figure S4.** The number of mutation genes and aberrantly methylated genes of each breast cancer sample among different subtypes. **Supplementary Table S1.** Pairwise comparisons for numbers of driven edges in ssMutat-DM, ssMethy-DM and co-driver modules respectively between subtypes. **Supplementary Table S2.** Pairwise comparisons for number of mutation genes, aberrantly methylated genes, hypermethylation genes and hypomethylation genes respectively between subtypes.

## Data Availability

All data analyzed in this study are publicly available. The sample-specific driver module construction method introduced in this study has been implemented in R, which is available at https://github.com/xumingmin/ssMutat-Methy--Driver-Modules.git.
