# Supplementary material for: Construction and analysis of sample-specific driver modules for breast cancer
Source: BMC Genomics. 2022 Oct 20;23:717. doi: 10.1186/s12864-022-08928-4 (PMC9583575; doi:10.1186/s12864-022-08928-4)
Supplement: Supplementary file 1 — Additional file 1: Supplementary Figure S1. The identified aberrantly methylated genes for each sample. Supplementary Figure S2. The boxplot of gene mutation frequency and gene methylation aberration frequency. Supplementary Figure S3. Sample-specific methylation aberration driver modules characterize personalized features and also reveal common network patterns for breast cancer samples. Supplementary Figure S4. The number of mutation genes and aberrantly methylated genes of each breast cancer sample among different subtypes. Supplementary Table S1. Pairwise comparisons for numbers of driven edges in ssMutat-DM, ssMethy-DM and co-driver modules respectively between subtypes. Supplementary Table S2. Pairwise comparisons for number of mutation genes, aberrantly methylated genes, hypermethylation genes and hypomethylation genes respectively between subtypes. [file 12864_2022_8928_MOESM1_ESM.docx]

**Supplementary Materials for**

**“Construction and analysis of sample-specific driver modules for breast cancer”**

**Overview of Supplementary Materials:**

**Supplementary Figures**

Supplementary Figure S1: The identified aberrantly methylated genes for each sample.

Supplementary Figure S2: The boxplot of gene mutation frequency and gene methylation aberration frequency.

Supplementary Figure S3: Sample-specific methylation aberration driver modules characterize personalized features and also reveal common network patterns for breast cancer samples.

Supplementary Figure S4: The number of mutation genes and aberrantly methylated genes of each breast cancer sample among different subtypes.

**Supplementary Tables**

Supplementary Table S1: Pairwise comparisons for numbers of driven edges in ssMutat-DM, ssMethy-DM and co-driver modules respectively between subtypes.

Supplementary Table S2: Pairwise comparisons for number of mutation genes, aberrantly methylated genes, hypermethylation genes and hypomethylation genes respectively between subtypes.

**Supplementary Figures**


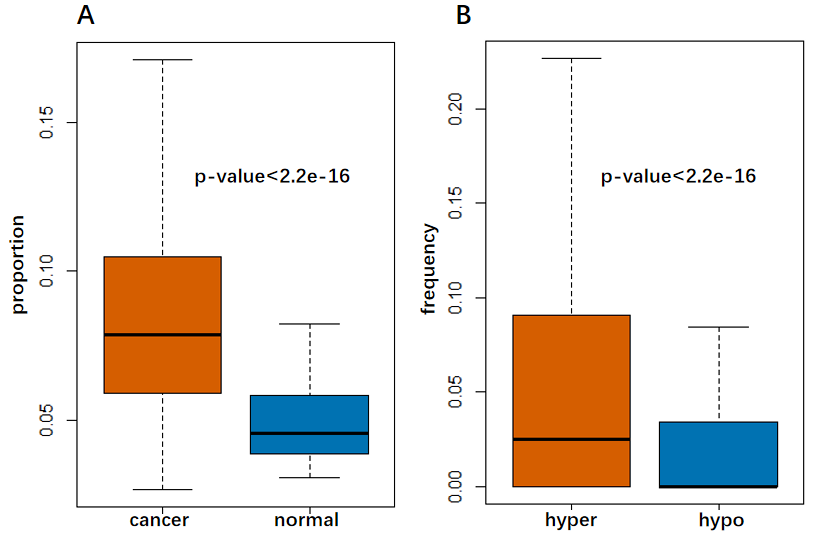


**Figure S1. The identified aberrantly methylated genes for each sample.** A, The proportion of aberrantly methylated genes in tumor and normal samples. B, The distribution of hypermethylation and hypomethylation frequencies for each gene in breast cancer samples.


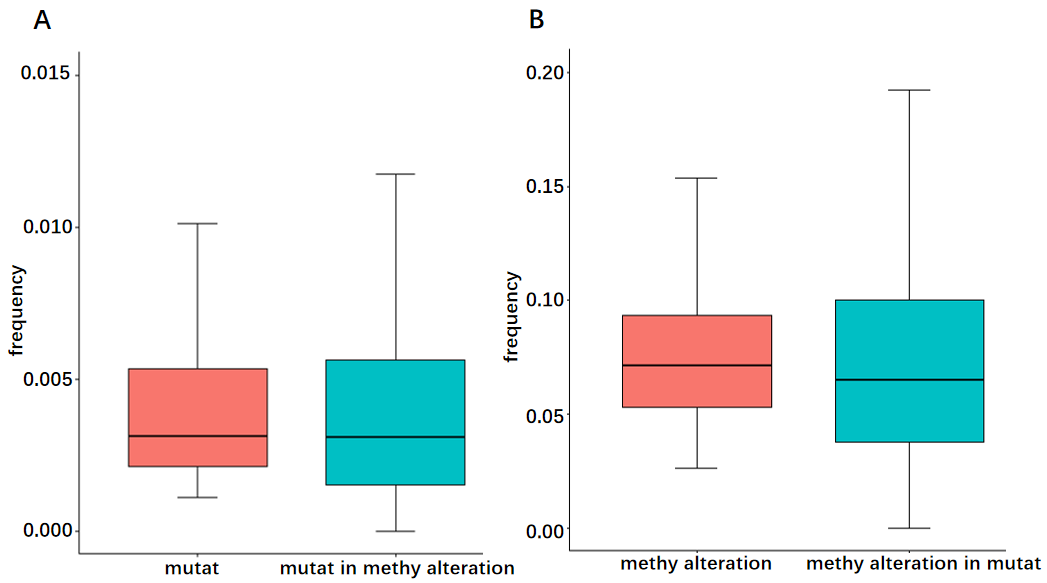


**Figure S2. The boxplot of gene mutation frequency and gene methylation aberration frequency.** A, The mutation frequency of all genes and of genes with methylation aberration. B, The methylation aberration frequency of all genes and of genes with mutation.


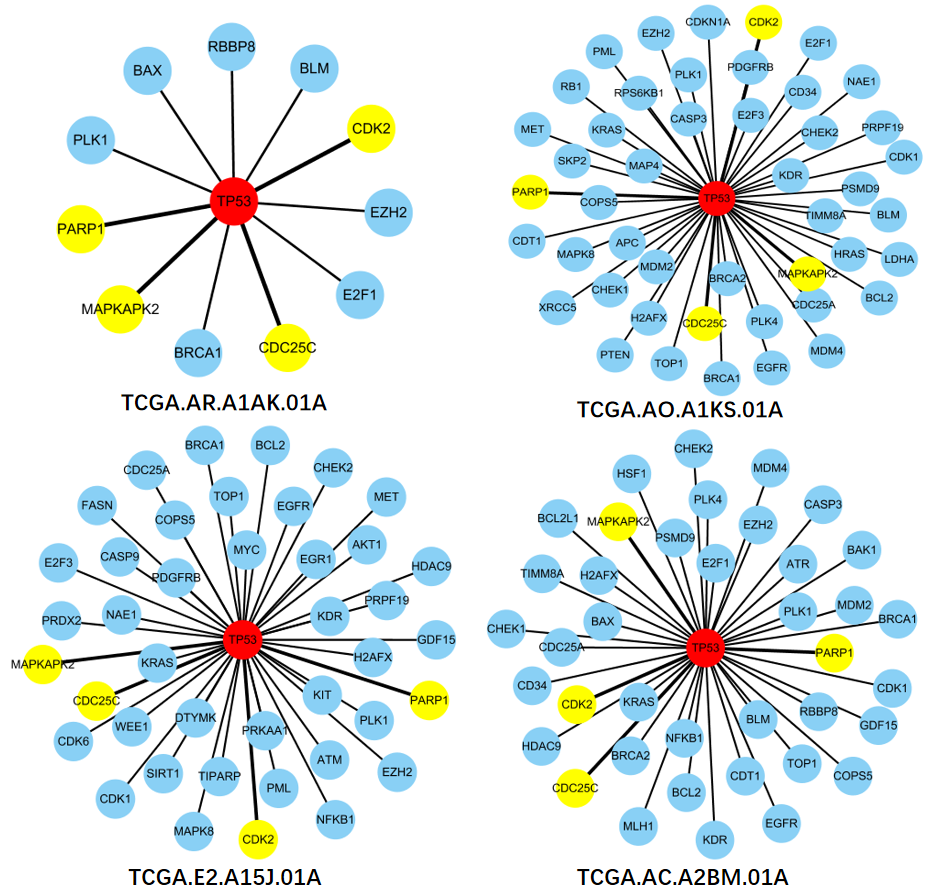


**Figure S3. Sample-specific methylation aberration driver modules characterize personalized features and also reveal common network patterns for breast cancer samples.** The four sample-specific methylation aberration driver subnetworks of gene TP53 from four samples of breast cancer. The numbers of the connections with TP53 for the four samples are respectively 12, 47, 42 and 40, and the genes linked to TP53 are different in the four breast cancer samples. However, PARP1, CDk2, CDC25C, and MAPKAPK2 (the yellow color) are common genes appearing in the four subnetworks.


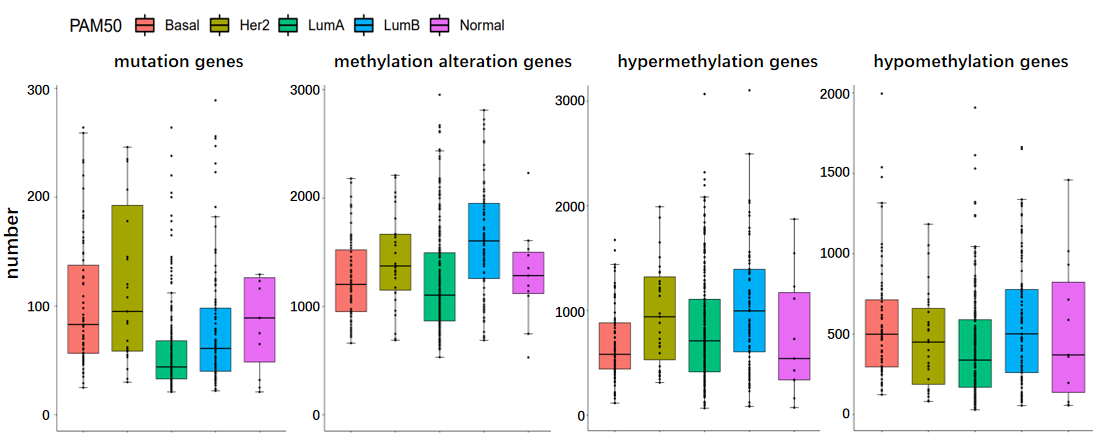


**Figure S4. The number of mutation genes and aberrantly methylated genes of each breast cancer sample among different subtypes.** The number of mutation genes (A), aberrantly methylated genes (B), hypermethylation genes (C) and hypomethylation genes (D) of each breast cancer sample among different subtypes.

**Supplementary Tables**

**Table S1.** **Pairwise comparisons for numbers of driven edges in ssMutat-DM, ssMethy-DM and co-driver modules respectively between subtypes.**

| **n.mutat.driver.edges** | **n.metha.driver.edges** | **n.codriver.edges** |
| --- | --- | --- |
| Her2 - Basal <0.0001 | Her2 - Basal <1e-04 | Her2 - Basal <0.0001 |
| LumA - Basal <0.0001 | LumA - Basal <1e-04 | LumA - Basal <0.0001 |
| LumB - Basal 0.0246 | LumB - Basal 1e+00 | LumB - Basal 0.9979 |
| Normal - Basal <0.0001 | Normal - Basal <1e-04 | Normal - Basal <0.0001 |
| LumA - Her2 <0.0001 | LumA - Her2 <1e-04 | LumA - Her2 <0.0001 |
| LumB - Her2 0.4549 | LumB - Her2 <1e-04 | LumB - Her2 <0.0001 |
| Normal - Her2 <0.0001 | Normal - Her2 <1e-04 | Normal - Her2 <0.0001 |
| LumB - LumA <0.0001 | LumB - LumA <1e-04 | LumB - LumA <0.0001 |
| Normal - LumA <0.0001 | Normal - LumA <1e-04 | Normal - LumA <0.0001 |
| Normal - LumB <0.0001 | Normal - LumB <1e-04 | Normal - LumB <0.0001 |

| **n.mutat.genes** | **n.methy.genes** | **n.hypermethy.genes** | **n.hypomethy.genes** |
| --- | --- | --- | --- |
| Her2-Basal 0.1102 | Her2-Basal 0.3637 | Her2-Basal 0.0364 | Her2-Basal 0.1359 |
| LumA-Basal <0.0001 | LumA-Basal 0.0128 | LumA-Basal 0.9994 | LumA-Basal <0.0001 |
| LumB-Basal 0.0717 | LumB-Basal <0.0001 | LumB-Basal 0.0006 | LumB-Basal 0.9978 |
| Normal-Basal <0.0001 | Normal-Basal 0.8367 | Normal-Basa l0.2224 | Normal-Basal 0.0507 |
| LumA-Her2 <0.0001 | LumA-Her2 <0.0001 | LumA-Her2 0.2840 | LumA-Her2 0.0007 |
| LumB-Her2 0.8503 | LumB-Her2 <0.0001 | LumB-Her2 0.1042 | LumB-Her2 0.1056 |
| Normal-Her2 0.0569 | Normal-Her2 0.5888 | Normal-Her2 0.4356 | Normal-Her2 1.0000 |
| LumB-LumA <0.0001 | LumB-LumA <0.0001 | LumB-LumA 0.0223 | LumB-LumA <0.0001 |
| Normal-LumA <0.0001 | Normal-LumA <0.0001 | Normal-LumA 0.6848 | Normal-LumA 0.0001 |
| Normal-LumB 0.9701 | Normal-LumB <0.0001 | Normal-LumB 0.0014 | Normal-LumB 0.0417 |

**Table S2. Pairwise comparisons for number of mutation genes, aberrantly methylated genes, hypermethylation genes and hypomethylation genes respectively between subtypes.**
